# A ferritin nanoparticle vaccine based on the hemagglutinin extracellular domain of swine influenza A (H1N1) virus elicits protective immune responses in mice and pigs

**DOI:** 10.3389/fimmu.2024.1361323

**Published:** 2024-05-21

**Authors:** Pan Tang, Enhui Cui, Jinghua Cheng, Benqiang Li, Jie Tao, Ying Shi, Jiajie Jiao, Enqi Du, Jingyu Wang, Huili Liu

**Affiliations:** ^1^ Institute of Animal Husbandry and Veterinary Science, Shanghai Academy of Agricultural Sciences, Shanghai, China; ^2^ Instrumental Analysis Center, Shanghai Jiao Tong University, Shanghai, China; ^3^ College of Veterinary Medicine, Northwest A&F University, Yangling, China

**Keywords:** nanoparticle vaccine, hemagglutinin extracellular domain, protective immunity, ferritin, swine influenza virus

## Abstract

**Introduction:**

Swine influenza viruses (SIVs) pose significant economic losses to the pig industry and are a burden on global public health systems. The increasing complexity of the distribution and evolution of different serotypes of influenza strains in swine herds escalates the potential for the emergence of novel pandemic viruses, so it is essential to develop new vaccines based on swine influenza.

**Methods:**

Here, we constructed a self-assembling ferritin nanoparticle vaccine based on the hemagglutinin (HA) extracellular domain of swine influenza A (H1N1) virus using insect baculovirus expression vector system (IBEVS), and after two immunizations, the immunogenicities and protective efficacies of the HA-Ferritin nanoparticle vaccine against the swine influenza virus H1N1 strain in mice and piglets were evaluated.

**Results:**

Our results demonstrated that HA-Ferritin nanoparticle vaccine induced more efficient immunity than traditional swine influenza vaccines. Vaccination with the HA-Ferritin nanoparticle vaccine elicited robust hemagglutinin inhibition titers and antigen-specific IgG antibodies and increased cytokine levels in serum. MF59 adjuvant can significantly promote the humoral immunity of HA-Ferritin nanoparticle vaccine. Furthermore, challenge tests showed that HA-Ferritin nanoparticle vaccine conferred full protection against lethal challenge with H1N1 virus and significantly decreased the severity of virus-associated lung lesions after challenge in both BALB/c mice and piglets.

**Conclusion:**

Taken together, these results indicate that the hemagglutinin extracellular-based ferritin nanoparticle vaccine may be a promising vaccine candidate against SIVs infection.

## 1 Introduction

Swine influenza (SI) is one of the most important respiratory diseases in pigs and leads to huge economic losses in the swine industry (1–3). Swine are influenza virus reservoirs that have caused outbreaks and pandemics and have raised major public health concerns worldwide (4). The typical clinical signs associated with SI infections in pigs are often characterized by rapid onset of pyrexia, coughing, labored breathing, loss of appetite, and lesions of pneumonia at slaughter (5). Although the mortality rates of SI infections are low, the morbidity rates can reach up to 100%. Vaccination remains the most effective and economical strategy for preventing and controlling influenza infections in both animals and humans.

Currently, licensed swine influenza vaccines available in China and around the world are mostly inactivated vaccines for monovalent or bivalent formulations; however, inactivated vaccines and their production have various limitations, including a shortage of SPF chicken embryos, low titers, adaptive mutations of viruses during serial passages, and long production cycles (6). Cell-based vaccines offer many advantages, including genetic stability, high titer, short cycle, and ease of development via good manufacturing practices (GMPs) (7–10).

Nanoparticles have emerged as important platforms for vaccine research in recent years. Nanoparticle vaccines can effectively deliver antigens to dendritic cells, which yield broader humoral and cellular immune responses than conventional vaccines (11). To date, a set of engineered nanoparticle vaccines has been successfully applied in the prevention and treatment for major diseases (12–15). Ferritin self-assembling nanoparticles (NPs) have recently been used in the design of SARS-CoV-2 and seasonal influenza vaccines, and it has been proven that the viral glycoproteins RBD and HA can be inserted into octahedral symmetry structures and trimerize properly (16, 17). The hemagglutinin protein is one of the two main surface glycoproteins of influenza virus, which mediates binding to receptors and subsequent membrane fusion, participates in the packaging and budding of virus particles, and is the main target molecule for vaccine design (18). In the previous study (19), we reported a novel nanoparticle vaccine HANP and evaluated its protective efficacy in BALB/c mice. HANP was a mixed micelle consisting of recombinantly expressed full-length swine influenza HA trimers that stably interact with the core of PS80 molecules through the hydrophobic TM domain. Here, we used HANP nanoparticle vaccine as control and compared it with another newly developed nanoparticle vaccine in mice.In the present research, a recombinant, baculovirus–insect cell system-expressed ferritin nanoparticle vaccine based on the HA extracellular domain of swine influenza virus was developed. After two immunizations, the immunogenicity and protective efficacy of the HA-ferritin nanoparticle vaccine against swine influenza virus in mice and piglets were evaluated.

## 2 Materials and methods

### 2.1 Viruses and cells

SIV A/swine/Qingdao/2018 (H1N1) HA sequences were downloaded from the GISAID Epiflu database with accession number EPI_ISL_370660. Two insect cell lines were used for producing the nanoparticle vaccine: Sf9 cells (Gibco™ Sf-900™ III SFM, Cat. No. 15236025, Carlsbad, USA) were used to propagate recombinant baculoviral stocks, and high five cells (BTI-TN-5B1–4, Cat. No. 10747474, Carlsbad, USA) were used to express the recombinant protein, and both were purchased by Xi’an Lihe Biotechnology Co., Ltd. (Xi’an, China) from Thermo Fisher Scientific (Waltham, MA, USA). Both cells were cultured in suspension in serum-free IB905 insect cell medium (World-Medium, Suzhou, China) at 28°C. Swine influenza virus H1N1 strain (CVCC AV1523) was purchased from the National Center for Veterinary Culture Collection (China Institute of Veterinary Drug Control, Beijing). HA protein sequences between A/swine/Qingdao/2018 (EPI_ISL_370660) and CVCC AV1523 (EPI_ISL_504932) in this study were quite different, with sequence homology of 81.8%, and they belong to different branches. The H1N1 strain was amplified at 37°C for 3 days in the allantoic cavities of 10-day-old SPF chicken embryos (Beijing Boehringer Ingelheim Life Biotechnology Co., Ltd., Beijing, China) and used in subsequent animal challenge studies and HAI tests.

### 2.2 Design and construction of nanoparticle vectors

**Figure 1:**
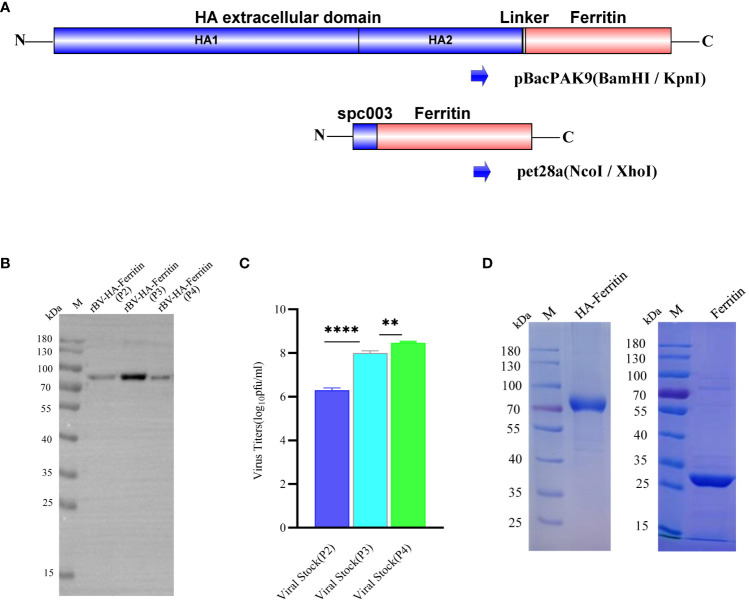
Analysis of the generation and expression characteristics of recombinant baculovirus. **(A)** Schematic of HA-ferritin and ferritin nanoparticle vector construction. **(B)** Western blots of protein expression of different recombinant baculovirus stocks. The molecular weight of HA-ferritin was approximately 80 kDa. **(C)** Viral titers of different generations of recombinant baculovirus stocks. (**p < 0.01, ****p < 0.0001). **(D)** SDS-PAGE analysis of purified HA-ferritin and ferritin nanoparticles; the molecular weights were approximately 80 kDa and 30 kDa, respectively.

Recombinant plasmid was designed and performed as previously reported (16). Briefly, I7E and N19Q mutations were introduced into *Helicobacter pylori* ferritin (GenBank NP_223316, residues 5–167) to abolish potential N-glycosylation site, the extracellular domain sequence of HA gene contains residues HA1 1-HA2 174, and all genes were Sf9 insect cells codon optimized and synthesized biochemically by GenScript (Nanjing, CN). HA-ferritin fusion genes were generated by fusing the ectodomain of HA with the ferritin gene by Ser-Gly-Gly linker and further cloned into the pBacPAK9 vector between the *Bam*HI/*Kpn*I sites downstream of a polyhedrin promoter for efficient expression (**Figure 1A**). Ferritin nanoparticles were used as a control, and the ferritin gene sequence (fused with a spycather003 protein) was optimized by a prokaryotic codon and inserted into the *Nco*I/*Xho*I sites of the pet28a vector, which was synthesized by GenScript (Nanjing, CN).

### 2.3 Protein biosynthesis and purification

To produce HA-ferritin nanoparticles, the recombinant plasmid was cotransfected into Sf9 cells with the qBac Bacmid containing Autographa californica multinuclear polyhedrosis virus genome and SofastTM Transfection reagent (Sunma, Xiamen, CN). P2–P4 viruses were obtained by the consecutive passage of P1 virus stock on Sf9 cells, the titers of recombinant baculovirus stocks were determined using a BacPAK™ baculovirus rapid titer kit (Clontech). Recombinant baculovirus was subsequently cultured on Hi5 cells, and the culture supernatants were collected 96 h post-transfection. The hemagglutination activity of the supernatant expressed by Hi5 cells was detected via an HA assay. The supernatant was concentrated and then filtered through a 0.45-µm filter membrane before purification. The HA-ferritin nanoparticles were purified by Capto Core 700 chromatography medium using a HiScale 26/20 column (Cytiva, Marlborough, MA) equilibrated with 25 mM Tris, 150 mM NaCl, pH 6.8. The flowrate was 2.0 ml/min, the flow-through was collected, and the column was further washed with the equilibrated buffer. The gel-filtration column was regenerated with 30% isopropanol and 1 M NaOH to desorb captured molecules, as recommended by the manufacturer. SDS-PAGE was performed to verify the size and purity of the recombinant HA-ferritin nanoparticles.

For ferritin nanoparticles, the recombinant plasmids were expressed in BL21 (DE3) *E. coli* and induced by IPTG at 37°C for 4 h. Cells were disrupted by sonication, and supernatant was clarified by centrifugation. Ferritin nanoparticles were purified by ion exchange chromatography (Buffer A: 20 mM Tris, pH 7.5; Buffer B: 20 mM Tris, 1 M NaCl, pH 7.5) on an HK16/20 Q Focurose HP column (Huiyan Biology) after heat treatment at 70°C for 20 min. The elution peaks were collected and analyzed via SDS-PAGE.

### 2.4 HA and HAI assays

The hemagglutination activity was determined via hemagglutination assays. In brief, 50 μl of PBS was added to the 96-well microplates, after which 50 μl of recombinant baculovirus expression suspension was added to the first column and diluted to column 11. Column 12 was used as a negative control. Finally, 50 μl of 0.5% chicken red blood cell suspension was added to all wells, which were subsequently transferred to a 25°C incubator for 30 min. The last dilution at which hemagglutination was observed was determined as the hemagglutination titer.

Hemagglutination inhibition (HAI) assays were performed as described previously (19). The swine influenza virus H1N1 (CVCCAV1523) strain was propagated in embryonated chicken eggs. Immune serum specimens were pretreated with receptor-destroying enzyme II (RDE II; Denka Seiken Co., Ltd.) at 37°C overnight and then 56°C for 45 min to inactivate the nonspecific enzyme, and HAI assays were performed in 96-well V-bottom plates using four HA units per well and 0.5% chicken red blood cells (RBCs). After an additional 30 min of incubation at room temperature, the HAI titer was defined as the reciprocal of the greatest serum dilution that inhibited virus hemagglutination of RBCs.

### 2.5 TEM and DLS analysis

The nanoparticles were further characterized via transmission electron microscopy (TEM) by Wuhan Servicebio Technology Co., Ltd. (Wuhan, China). Briefly, purified HA-ferritin nanoparticles were added to a carbon-coated copper grid for 60 s, after which the samples were negatively stained with phosphotungstic acid and ammonium molybdate, and images were recorded using a Hitachi 7000 TEM system (Hitachi High-Technologies, Ibaraki, Japan) at an accelerating voltage of 80 kV.

The hydrodynamic diameter (Z-average size) of influenza HA-ferritin nanoparticle was measured by dynamic light scattering (DLS) using a Zetasizer Nano ZS instrument (Malvern Instruments Limited, Worcestershire, UK). Particles were dispersed using sterile phosphate-buffered saline at a concentration of 1 mg/ml. The Z-average is represented as diameter in nm ± width of the distribution.

### 2.6 Formulation of vaccines

HA-ferritin nanoparticles were administered at a dose of 10 µg of nanoparticle protein in 0.2 ml for BALB/c mice and 50 µg protein in 2 ml for piglets, respectively. MF59 adjuvant was stored and supplied by Yangling Kerry Biotechnology Co., Ltd. and mixed with nanoparticle vaccine at a 1:1 ratio. The inactivated influenza vaccine(IIV) used in this study contained 10^7.2^ EID50/0.1 ml of formaldehyde-inactivated SIV of H1N1 (CVCCAV1523) strain. HANP, a non-spherical nanoparticle subunit vaccine with a core of PS80 micelles, was reported in a previous study by our team (19).

### 2.7 Immunity and challenge experiment of mice

Six-week-old female BALB/c mice (n = 48) were purchased from Vital River Laboratory Animal Technology Co., Ltd. (Beijing, China) and divided randomly into six groups. Mice were maintained in IVC systems (Fengshi Group, Suzhou, China) and provided with *ad libitum* water and feed. The room relative humidity was maintained at 50% ± 10%, the temperature was maintained at 22°C ± 3°C, and supplied with a 12-h on/off photoperiod. To determine the immunogenicity of HA-ferritin nanoparticle vaccine, mice were prime immunized subcutaneously with 10 μg of HA-ferritin protein with or without MF59 adjuvant, 10 µg of ferritin protein, 0.2 ml of inactivated influenza vaccine (IIV), and 10 µg of HANP subunit vaccine. The negative control group was treated with PBS. The injection volume was 0.2 ml per mouse, and mice were received a booster immunization in the same manner after 3 weeks.

**Figure 2:**
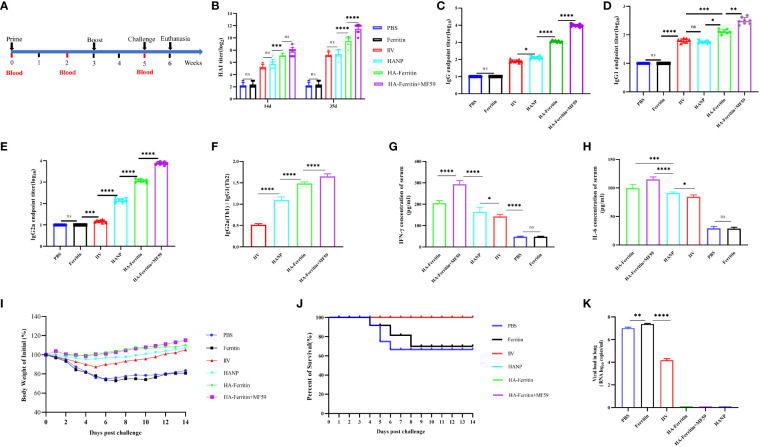
Protective immunity induced in mice immunized with the HA-ferritin nanoparticle vaccine. **(A)** Schematic flow diagram of the animal immunization and challenge. **(B)** HAI antibody titers in mice immunized with different vaccines on 14 days and 35 days. **(C)** The endpoint titer of HA-specific IgG in serum 2 weeks after the boost immunization. Optical densities were analyzed at 450 nm. **(D–F)** The endpoint titer of HA-specific IgG1 and IgG2a in the serum of immunized mice 2 weeks after boost immunization. Each symbol represents an individual mouse, and the horizontal bar indicates the geometric mean of the group. **(G, H)** IFN-γ and IL-6 levels in the serum of immunized mice were analyzed by mice IFN-γ and IL-6 enzyme immunoassay kit. **(I)** Weight changes in mice challenged with swine influenza virus H1N1 in each group are shown. Values indicate the mean weight changes of all mice in each group after virus challenge. **(J)** Survival rates were analyzed by Kaplan–Meier method (n = 8/group). **(K)** Viral loads in the lung tissues of mice six days post challenge with swine influenza virus H1N1 are shown. The data was expressed as the means ± SEM. (ns, not significant, *p < 0.05, **p < 0.01, ***p < 0.001, ****p < 0.0001).

Serum samples were collected from all mice at the indicated time points after each immunization (**Figure 2A**). Mice were challenged intranasally (i.n.) with 10^7.2^ EID50 of swine influenza H1N1 strains 0.1 ml at week 5. The immune effect was evaluated comprehensively by clinical observation, body weight curve, viral load, and histopathological examination in the lung, and the relevant detailed indicators and evaluation criteria can refer to the previously published article (19). On the sixth day post-challenge, three mice in each group (including mice that died naturally) were euthanized and dissected, and the lung tissues were taken for hematoxylin–eosin (HE) staining and viral load detection, while the other five mice were observed and recorded until the 14th day. The primers and probes used for RT-qPCR were as follows: forward primer, 5′-CCMAGGTCGAAACGTAYGTTCTCTCTATC-3′; reverse primer, 5′-TGACAGRATYGGTCTTGTCTTTAGCCAYTCCA-3′; and probe, 5′-FAM-ATYTCGGCTTTGAGGGG GCCTG-MGB-3′.

### 2.8 Immunity and challenge experiment of piglets

A total of 20 4-week-old conventionally weaned piglets were purchased from a commercial farm. All piglets were negative for the SIV antibody according to serological testing and antigen negative for SIV, PRRSV, PCV2, ASFV, and PEDV according to polymerase chain reaction (PCR). Piglets were randomly divided into four groups (five pigs in each group) and provided free access to feed and drinking water, the barn was cleaned twice a day, and the pig manure was removed.

Piglets in the negative control group were injected intramuscularly with 2 ml of PBS. Swine-influenza-inactivated vaccine group was immunized intramuscularly with 2 ml of IIV. Piglets in the nanoparticle groups were injected intramuscularly with 2 ml of HA-ferritin or MF59-adjuvanted HA-ferritin vaccine, and both groups contained 50 µg of nanoparticle protein. Piglets were immunized on Day 0 and boosted on Day 21, and 14 days following the prime and boost immunizations, the serum was collected for antibody detection.

Piglets in the immune groups were challenged intranasally (i.n.) with 10^7.2^ EID50/0.1 ml of swine influenza H1N1 strain (2ml) at 35 days, while those in the PBS group were divided into two subgroups, three (3/5) were challenged, and two (2/5) were used as healthy negative controls (NC). Following the challenge, piglets were monitored daily for rectal temperature and clinical signs. Fever was defined as a rectal temperature ≥40.5°C. Piglets were euthanized humanely 6 days post-challenge, and lung samples were collected for viral load analysis and further histopathological examination. One gram of lung tissue was added to 1 ml PBS and fully ground by a homogenizer, and 200 μl of each sample was taken for nucleic acid extraction and RT-qPCR detection. Nasal swabs were collected before challenge and daily after challenge until the piglets were sacrificed; real-time PCR was performed, and viral loads were quantified.

### 2.9 Antigen-specific IgG and cytokine assays

Serum-specific IgG and IgG subclass (IgG1 and IgG2a) levels in immunized mice and piglets (only IgG) were detected by enzyme-linked immunosorbent assay (ELISA) as described previously (20). Briefly, the plates were coated with 4 μg/ml of recombinant HA-ferritin proteins and incubated overnight at 4°C. Plates were then blocked with 5% skim milk at 37°C for 1 h. Sera were serially diluted and added to the wells for 1 h. Horseradish peroxidase (HRP)-conjugated goat anti-mouse IgG (Biosharp, Beijing, China, Catalog BL001A, used 1/5,000), mouse IgG1 (Proteintech, Wuhan, China, Catalog SA00012–1, used 1/5,000), and mouse IgG2a (Proteintech, Wuhan, China, Catalog SA00012–2, used 1/5,000) antibody were used to detect each isotype. Plates were washed, and TMB substrate was applied; reaction was stopped with 1M H_2_SO_4_. The absorbance value of 450 nm wavelength was recorded by a microwell plate analyzer (Prolong, DNM-9602; Beijing). The levels of IFN-γ and IL-6 in mouse serum samples and IFN-γ in pig serum samples were measure by ELISA using the enzyme immunoassay kits (MlBio, Shanghai, China) according to the manufacturer’s instructions.

### 2.10 Histopathology

The pathological analysis of lung tissue samples was completed by LiLai Medical Laboratory Center (Chengdu, Sichuan) according to the standard operating procedure, and the histopathological changes and scores of different lung tissues were recorded in detail. The tissue sections were scanned with a Pannoramic 250 digital slice scanner (3DHistech, Budapest, Hungary).

### 2.11 Statistical analysis

All the experimental data are presented in mean ± standard deviation (SD), and the statistical analyses were performed by GraphPad Prism 9.0 software. The significant differences between two groups were performed by one-way ANOVA with Tukey’s multiple comparisons test, and differences were statistically significant when *p*-values <0.05.

### 2.12 Ethics statement

All animal experiments were performed in accordance with the guidelines of the Animal Welfare Council of China and were approved by the Ethical Committee for Animal Care and Use of Northwest A&F University. During the whole experiment, the 3R principle (Reduction, Replacement, and Refinement) was strictly applied. The number of mice and piglets used in the experiment was strictly controlled, and anesthesia was carried out before dissection to ensure animal welfare.

## 3 Results

### 3.1 Generation of recombinant baculovirus expressing HA protein

Hemagglutinin (HA) is a major surface glycoprotein of influenza virus and is often used as the main target for vaccine research. To further optimize the HA-ferritin nanoparticle vaccine, the extracellular domain sequence of the HA gene was fused to *H. pylori* ferritin, and the fusion fragment was inserted into the pBacPAK9 vector. A schematic diagram is shown in **Figure 1A**.

To verify the proliferation of recombinant baculovirus and the expression of HA-ferritin fusion protein in Sf9 cells, virus titers were determined and Western blotting analyses were performed. Western blotting analysis of recombinant proteins was performed after samples were resolved by SDS-PAGE and transferred onto PVDF membranes. Recombinant baculovirus was further expressed in Hi5 cells, and the hemagglutination activity was analyzed by hemagglutination assay. As shown in **Figure 1B**, the HA-ferritin fusion protein expressed in the culture of different recombinant baculovirus stocks of passage 2 (P2), passage 3 (P3) and passage 4 (P4) of Sf9 was specifically detected by HA antibody (Sino Biological, Cat: 11085-T62, Beijing), indicating that the recombinant baculovirus rBV-H1-HA-ferritin was successfully constructed.

To confirm the replication of recombinant baculovirus, viral titer was determined by using a BacPAK™ Baculovirus Rapid Titer Kit. Results showed that viral titration of recombinant baculovirus in Sf9 cells increased gradually with the number of passages (**Figure 1C**). To obtain the purified HA-ferritin fusion protein, High Five insect cells were infected with the recombinant baculovirus stock P4; after 96 h of infection, recombinant baculovirus was harvested. Hemagglutination assay showed that the hemagglutination titer of recombinant baculovirus supernatant expressed in Hi5 cells was 1:8192(2^13^). HA-ferritin fusion protein was purified by Capto core700 anion exchange resin in an SDL-100 purifier system (Sepure instruments, Suzhou) and ultimately analyzed via SDS-PAGE (**Figure 1D**, left). Single ferritin protein was expressed and purified from BL21 *E. coli* cells and confirmed by SDS-PAGE (**Figure 1D**, right). The results revealed that the purified HA-ferritin fusion protein and ferritin had high purities, and the molecular weights were approximately 80 kDa and 30 kDa, respectively, which were consistent with expectations.

### 3.2 Characterization of HA-ferritin nanoparticles

**Figure 3:**
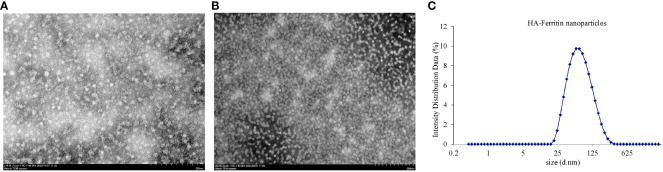
Transmission electron microscopy (TEM) analysis of HA-ferritin nanoparticles **(A)** and ferritin nanoparticles **(B)**, dynamic light scattering (DLS) analysis of HA-ferritin nanoparticles **(C)**.

To evaluate the physical properties of the HA-ferritin nanoparticles, TEM analysis was used for identifying the morphology of the HA-ferritin and DLS analysis was used for diameter. TEM analysis of the concentrated nanoparticle protein revealed that both ferritin and HA-ferritin truly self-assembled into nanoparticles with high density and with diameters of approximately 40 nm and 15 nm (**Figures 3A, B**). Compared with those of smooth, spherical ferritin nanoparticles, protrusions that probably exhibited variable HAs were observed on the surface of HA-ferritin nanoparticles. These results are consistent with a previous report (16). DLS analysis showed that the particle size of HA-ferritin nanoparticle mostly ranged from 32.74 nm to 52.06 nm in diameter (**Figure 3C**), which is consistent with the TEM result.

### 3.3 HA-ferritin vaccination induces protective immune responses in BALB/c mice

The immunization and challenge of the mice were carried out according to the experimental scheme (**Figure 2A**). To assess the immunogenicity of the HA-ferritin nanoparticle, mice were immunized twice with HA-ferritin nanoparticles with or without MF59 adjuvant. IIV and HANP subunit vaccine were immunized as conventional vaccine control groups, while ferritin nanoparticles and PBS were used as negative controls. Our results showed that HA-ferritin nanoparticle induced higher titer of hemagglutination inhibition (HAI) antibodies than HANP and IIV. On day 14, the HAI titer of HA-ferritin group was 2^8^, while that of IIV and HANP group was only 2^6^; on day 35, the HAI titer of HA-ferritin group was 2^10^, while the HAI titer of IIV and HANP group was only 2^8^ (**Figure 2B**). The HAI antibody titers of HA-ferritin arrived at 2^9^ 2 weeks after the boost immunization, which indicated a strong immunogenicity, whereas the HAI titers of the HANP and IIV groups reached only 2^7^ at the same time point. MF59 adjuvant can enhanced the HAI titers of HA-ferritin; although there was no significant difference between HA-ferritin and MF59-adjuvanted HA-ferritin group at 14 days (2 weeks after prime immunization), there was a significant difference at 35 days (2 weeks after boost immunization), and the average HAI titer of the MF59-adjuvanted HA-ferritin group was 2^12^, while HA-ferritin, HANP and IIV groups were 2^10^, 2^8^, and 2^8^, respectively. No HAI antibodies were detected in ferritin or PBS groups.

To further illuminate the models of the humoral immune response conducted by nanoparticle vaccines, serum titers of HA-specific total IgG and its subclasses of IgG1 and IgG2a levels were measured by enzyme-linked immunosorbent assay (ELISA). The results indicated that mice vaccinated with HA-ferritin produced strong HA-specific IgG responses, while mice that received PBS or ferritin were negative for HA-specific IgG (**Figure 2C**). MF59 enhanced HA-specific IgG and IgG subclass responses. HA-ferritin and MF59-adjuvanted HA-ferritin groups exhibited a more significant IgG and IgG subclass antibodies than HANP and IIV groups (**Figures 2D, E**), and the ratio of IgG2a/IgG1 in nanoparticle vaccine groups demonstrated a higher IgG2a level compared with IgG1, indicating Th1-favored responses, whereas HANP and IIV groups tended to a Th2 type immune responses (**Figure 2F**).

Cytokines are important in the establishment of innate immunity and in determining the magnitude and dimension of immune responses. Concentrations of cytokines in serum were evaluated 2 weeks after boosting immunization by ELISA. As shown in **Figure 2G**, IFN-γ levels in HA-ferritin and MF59-adjuvanted HA-ferritin groups increased compared with the HANP and IIV groups. A similar trend was observed in the IL-6 analysis (**Figure 2H**).

We then evaluated the protective effect of the HA-ferritin nanoparticle vaccine against live swine influenza virus infection. Two weeks after the boost immunization, mice were challenged with 10^7.2^ EID50 of swine influenza H1N1 virus. Physiological indicators of mental state, weight loss, and viral loads were monitored following the challenge. No significant weight fluctuations were found in mice immunized with MF59-adjuvanted nanoparticles group, mice in HA-ferritin group suffered less weight loss compared to the HANP and IIV-vaccinated groups (**Figure 2I**), whereas the mice in the PBS and ferritin-inoculated groups experienced severe weight loss of >20%. The survival rates of the various groups were recorded over time and illustrated using the Kaplan–Meier method (**Figure 2J**).

**Figure 4:**
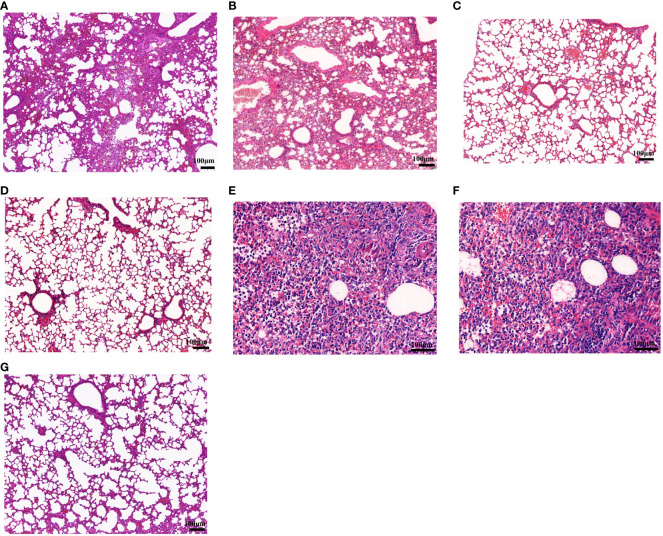
Histopathological examination of lung lesions in mice on the sixth day post-challenge. **(A–F)** Lung changes in the different groups. **(A)** Inactivated vaccine (IIV) group; **(B)** HANP group; **(C)** HA-ferritin group; **(D)** HA-ferritin + MF59 group; **(E)** PBS group; **(F)** ferritin group; **(G)** NC (scale bar = 100 µm).

At 6 days post-challenge (dpc), mice were killed by CO_2_ inhalation, and the viral loads and pathological changes in lungs were evaluated. HA-ferritin nanoparticle vaccine can significantly reduce viral load in challenged mice, and no virus was detected in mice vaccinated with HA-ferritin and HANP, while mice receiving IIV showed a lower viral loads of 6.4 × 10^4^ copies/ml. In contrast, mice inoculated with the PBS or ferritin showed high loads of infectious virus in lung tissues, with viral loads of 5.4 × 10^7^ copies/ml and 6.8 × 10^7^ copies/ml, respectively (**Figure 2K**). Lung pathological changes of IIV vaccine group are shown in **Figure 4A**. The lung tissue structures in mice vaccinated with HANP and IIV were basically normal; only slight histopathological changes were found (**Figure 4B**). No obvious pathological changes were observed in mice vaccinated with HA-ferritin or MF59-adjuvanted HA-ferritin groups (**Figures 4C, D**). In contrast, the PBS- or ferritin-challenged group exhibited alveolar wall congestion, alveolar epithelial cell necrosis, cell structure disintegration, accompanied by the slight proliferation of alveolar epithelial cells and more inflammatory cells were infiltrated, showing typical symptoms of interstitial pneumonia after challenge (**Figures 4E, F**). The lung pathological scores of different groups are shown in **Table 1**. HE analysis of mice in the normal control (NC) group was shown in **Figure 4G**.

**Table 1:** Pathological scores of mouse lungs after challenge.

Groups	Results
HA-ferritin	(–)
HA-ferritin+MF59	(–)
IIV	Inflammatory cell infiltration (+), thickening of alveolar septum (+)
HANP	Thickening of alveolar septum (+)
PBS-challenged	Alveolar structures disappeared (+++) Alveolar epithelial cell proliferation (++) Inflammatory cell infiltration (++++)
Ferritin-challenged	Alveolar structures disappeared (+++) Alveolar epithelial cell proliferation (++) Inflammatory cell infiltration (++++)

No lesions, (−); lesions, minimal (+), mild (++), moderate (+++), and severe (++++).

Taken together, these findings demonstrated the favorable protective efficacy of HA-ferritin in mice.

### 3.4 HA-ferritin elicits protective immune responses and protects pigs against SIV infection

The immunization and challenge scheme used for the piglets was the same as that used for the mice. In brief, all piglets were vaccinated twice at week 0 and week 3. HAI titer, total IgG concentration, and IFN-γ were detected. Following challenge with the SIV of H1N1 strain at week 5, rectal temperatures, macroscopic lung lesions, and viral loads in the lungs and nasal swabs of pigs were recorded.

**Figure 5:**
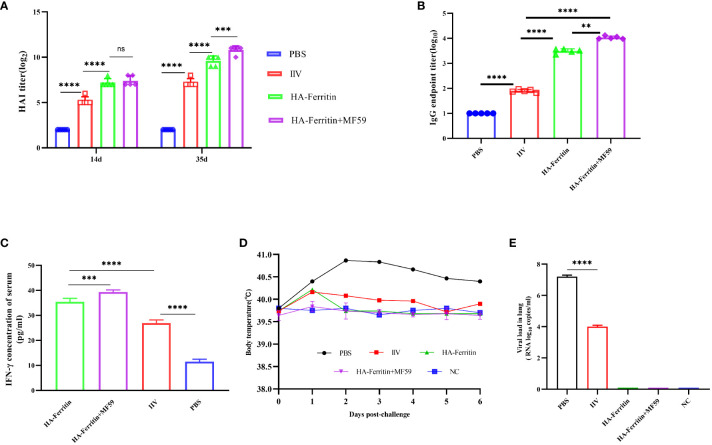
Protective immunity induced in piglets immunized with the HA-ferritin nanoparticle vaccine. **(A)** HAI antibody titers in piglets immunized with HA-ferritin, MF59-adjuvanted HA-ferritin, or IIV. **(B)** The endpoint titer of HA-specific IgG in the serum 2 weeks after boost immunization. Each symbol represents an individual piglet, and the horizontal bar indicates the geometric mean of the group. **(C)** IFN-γ levels in serum of immunized piglets were analyzed by pig IFN-γ enzyme immunoassay kit. **(D)** Mean rectal temperatures of immunized piglets after challenge with swine influenza virus H1N1. **(E)** Viral loads in lung tissues of piglets six days post challenge with swine influenza virus H1N1 are shown. The data was expressed as the means ± SEM (n = 5; ns, not significant, **p < 0.01, ***p < 0.001, ****p < 0.0001).

The HAI antibody titers of piglets in the PBS group were negative, while the HAI titers in the HA-ferritin group, MF59-adjuvanted HA-ferritin group, and IIV group increased with time (**Figure 5A**). At 14 dpc, the HAI titers of the HA-ferritin group and MF59-adjuvanted HA-ferritin group were 7 log2, while that of the IIV group was 5 log2. At 35 dpc, the HI antibody titers of the above three groups increased to 9 log2, 10 log2, and 7 log2, respectively. HA-specific total IgG detection in piglets revealed that all the vaccines induced specific IgG antibodies, but the IgG titers in the HA-ferritin group and MF59-adjuvanted HA-ferritin group were significantly higher than that in the IIV group (**Figure 5B**).

IFN-γ plays an important role in influenza antiviral immunity. In this study, the level of IFN-γ in the HA-ferritin group was significantly higher than that in IIV and PBS groups, and IFN-γ level in the MF59-adjuvanted nanoparticle vaccine group was significantly higher than the HA-ferritin group (**Figure 5C**).

**Figure 6:**
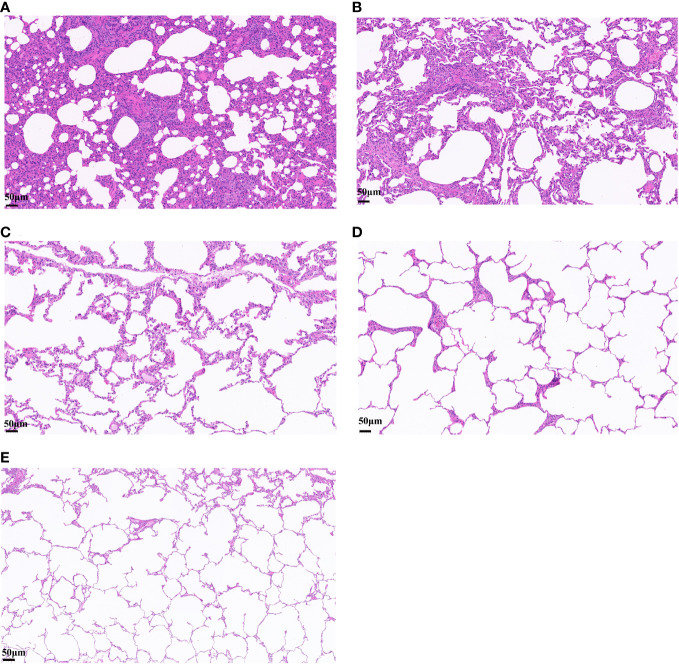
Histopathological analysis of lung lesions in piglets infected with swine influenza virus H1N1. **(A–E)** Lung changes in different groups. **(A)** PBS-challenged group. **(B)** Inactivated vaccine (IIV) group. **(C)** HA-ferritin group. **(D)** HA-ferritin+MF59 group. **(E)** NC (scale bar = 50 µm).

After challenge, obvious symptoms of mental depression, lethargy, and anorexia were observed. Moreover, there was a significant difference in mean rectal temperature between the PBS-challenged pigs and HA-ferritin or IIV-vaccinated groups from 2 to 6 days post-challenge, while there were no significant differences among the HA-ferritin, MF59-adjuvanted HA-ferritin, and IIV groups (**Figure 5D**). Specifically, the group of PBS-challenged pigs had fever of approximately 40.2°C–41°C on Days 2 to 6, and on Day 5, two pigs developed suspected cough and asthma symptoms. No obvious clinical symptoms were observed in other groups during the trial. Histopathological examination of lung lesions in piglets at 6 dpc is shown in **Figure 6**, and the corresponding pathological scores are shown in **Table 2**. Minimal thickening of the alveolar septum was found in the IIV group (**Figure 6B**), and mild alveolar epithelial cell proliferation and moderate inflammatory cell infiltration were observed in lungs of the PBS-challenged pigs (**Figure 6A**). No significant pathological lesions were found in the lungs of HA-ferritin-immunized groups (**Figures 6C, D**). Analysis of viral load in lungs showed that no virus was detected in piglets immunized with nanoparticle vaccine at 6 dpc but with a viral load of 3.5×10^4^ copies/ml in the IIV group (**Figure 5E**). In contrast, the viral load in PBS-challenged piglets was 7.2×10^7^copies/ml; the results were consistent with the analysis of histopathologic examination. No virus was detected in the nasal swabs of piglets immunized with nanoparticle vaccine or IIV, while the virus was detected in the PBS-challenged group on the third to fifth days, and the viral loads were 2.6×10^7^ copies/ml, 3.3×10^7^copies/ml, and 4.1×10^7^ copies/ml, respectively.

**Table 2:** Pathological scores of lungs of piglets after challenge.

Groups	Results
HA-ferritin	(−)
HA-ferritin+MF59	(−)
IIV	Thickening of alveolar septum (+)
PBS-challenged	Alveolar epithelial cell proliferation (++) Inflammatory cell infiltration (+++)

No lesions, (−); lesions, minimal (+), mild (++), moderate (+++).

## 4 Discussion

The occurrence and prevalence of swine influenza have caused great harm to both the pig industry and the public health system (1–3). Vaccination remains the most effective and economical strategy to prevent and control influenza virus infections in animals and humans. At present, commercial swine influenza vaccines at home and abroad are mainly inactivated vaccines, which have obvious limitations, such as the low antibody levels, the supplement of chicken embryos, and the replacement of epidemic strains (21). Nanoparticle vaccines display multiple viral antigenic components on the skeletons and induce both humoral and cellular immune responses, and have shown promising applications in the prevention and treatment of major diseases such as seasonal influenza, COVID-19, hepatitis B, AIDS, and tumors (16, 17, 22–25).

In the present study, we constructed a recombinant baculovirus expressing HA-ferritin fusion protein. Western blotting showed that the fusion protein HA-ferritin was efficiently expressed in Sf9 cells. Hemagglutination assays showed that the hemagglutination titer of recombinant baculovirus supernatant expressed in Hi5 cells was up to 2^13^. TEM analysis revealed that HA-ferritin can assemble into nanoparticles with high density and a diameter of approximately 25 nm.

We then evaluated the efficacy of the recombinant HA-ferritin nanoparticle vaccine against a different H1N1 SIV strain; the homology of HA sequence between the vaccine strain and the challenge strain was 81.8%. Immunization of mice and piglets with HA-ferritin nanoparticle vaccine induced powerful humoral and cellular immune responses and reduced lung lesions after SIV challenge. We detected HA-specific IgG and IgG subclass (IgG1 and IgG2a) antibodies in mice and piglets (only IgG) immunized with HA-ferritin or IIV vaccine by ELISA, and the IgG levels of both mice and piglets vaccinated with HA-ferritin were remarkably higher than those receiving IIV vaccine 2 weeks after the boost immunization, indicating that the HA-ferritin nanoparticle vaccine induced a strong humoral response. Moreover, the levels of IgG subclass (IgG1 and IgG2a) antibodies in mice and their ratios demonstrated Th1-favoring responses, which represented a strong level of cellular immunity. In our previous report (19), we studied the type of immune response of HANP in mice, and HA-ferritin vaccine in the present study produced higher and more comprehensive levels of humoral and cellular immunity than HANP.

A novel vaccine construction method that displays antigen components through self-assembling nanoparticle skeleton has become a hot topic in vaccine research and development in recent years (26). Pan (27) reported a Nano-B5 platform for one-step synthesis of nanoparticle vaccines in organisms, which achieved the modular assembly of nanoskeletons and antigens and provided a new idea for the rapid construction of novel vaccines. In addition, Ma (28) constructed two COVID-19 nanoparticle vaccines based on ferritin, which were coupled to the RBD region and HR regions of S protein. Animal experiments showed that these two nanoparticle vaccines induced stronger protective immunity than the conventional subunit vaccine in both mice and rhesus monkeys; the design and construction ideas are consistent with ours in this study, both achieving ideal protective immune effects. Ma (29)developed a nanoparticle vaccine GP5m-ferritin against PRRSV, piglets experiment showed that GP5m-ferritin vaccine elicited higher serum antibody titers in pigs than inactivated PRRSV, and GP5m-ferritin-vaccinated group had significantly lower mean rectal temperatures, respiratory scores, viremia, and macroscopic and microscopic lung lesion scores post-challenge compared with unvaccinated pigs. In our study, HA-ferritin-vaccinated piglets showed significantly less lung viral loads and nasal shedding, and fewer lung pathological lesions than those in PBS and inactivated vaccine groups. The results in our study broaden the application field of novel nanoparticle vaccines once again and prove that nanoparticle vaccines are promising vaccine candidates.

Cytokines play an important role in immune regulation and anti-infection, and their expression level is affected by many factors, including vaccine type, inoculation dose, and individual immune status. Referring to the previous research on nanoparticle vaccine, we focused on the changes in IFN-γ and IL-6 levels after immunization (16, 30). We found that the level of IFN-γ in the nanoparticle group was significantly higher than that in the non-immunized group, which may represent a stronger level of cellular immunity. IL-6 is a multifunctional cytokine, which plays an important role in humoral immunity and cellular immunity. In this study, the level of IL-6 in nanoparticle group was significantly upregulated compared with unimmunized group and inactivated vaccine group, which may be consistent with the higher level of cellular and humoral immunity to some extent. In addition, IL-6 is also involved in the process of inflammation and fever. In this study, we did not pay attention to the changes in IL-6 and other representative cytokines such as IL-2, IL-4, and TNF-α in mice after challenge, and we will analyze these in our subsequent study.

The viral load in the lung tissue is higher than that in nasal swabs, which may be related to the infection process of SIV. Combined with clinical symptoms, 4 days–6 days may be the most serious infection time in piglets after challenge. The lungs are the main target organs of SIV, and the replication and accumulation of SIV in the lung may be more significant. In addition, it may also be related to the shallow position of the swab inserted into the nasal cavity during sampling.

MF59 is a safe and potent vaccine adjuvant that has been licensed in more than 30 countries since its first approval in Europe in 1997 (31–33). MF59 has been used in combination with the seasonal flu vaccine Fluad^®^ (34–36), which elicits a stronger immune response in different populations, including elderly individuals and children. In the present research, MF59 adjuvant significantly promoted both humoral and cellular immunity compared with other groups and had a better protective effect after challenge. Moreover, self-assembled ferritin also functions as an adjuvant, not just as an antigen display platform; the mechanism is still unclear and needs to be further studied.

Swine influenza virus mainly infects pigs through the surface of the respiratory mucosa, so inducing an efficient mucosal immune response is also an important goal for novel vaccine development. Pan (37) successfully developed a new candidate broad-spectrum nanoparticle vaccine, HMNF, against influenza virus, which can induce a high titer of antigen-specific antibody and high level of cellular immune response after nasal mucosa immunization, and achieved cross-protection against multiple influenza A and B virus subtypes. Mucosal immunity will also be our goal in the next phase of validation. In addition, previous studies (38) have proven that nanoparticle vaccines produce longer-lasting antibody levels than conventional subunit vaccines and inactivated vaccines. In this study, we monitored the antibody level only until the 35th day post-immunization, and whether HA-ferritin nanoparticles can also stimulate longer-lasting protection than conventional vaccines needs to be further proven. At the same time, due to the limitations of the virus source and biosafety, this study verified the protective effect of HA-ferritin on only one circulating SIV strain, and whether it can achieve cross-protection between different SIV strains will be further proven.

Although no death occurred in the piglets after virus attack in our study, we observed obvious symptoms of mental depression, lethargy, and anorexia. Clinically, SI infection often has a low mortality rate, but it often leads to decreased feed utilization and poor growth performance and may cause co-infection with other pathogens and cause high morbidity and even death. Therefore, the clinical infection and morbidity of swine flu should arouse attention.

The vaccine strain and the challenge strain used in this study are not the same strain, and their HA sequence homology is 81.8%, which may indicate that the nanoparticle vaccine developed in this study has the potential of cross-protection against different strains. Due to the limited source of SIV strains, we did not verify the cross-protection of nanoparticle vaccines against different SIV strains, and we will supplement in our follow-up studies.

## Data availability statement

The datasets presented in this study can be found in online repositories. The names of the repository/repositories and accession number(s) can be found in the article/**Supplementary Material**.

## Ethics statement

The animal studies were approved by the institutional laboratory of the Animal Care and Use Committee of Northwest A&F University. The studies were conducted in accordance with the local legislation and institutional requirements. Written informed consent was obtained from the owners for the participation of their animals in this study.

## Author contributions

PT: Formal analysis, Methodology, Project administration, Validation, Writing – original draft. EC: Methodology, Software, Writing – original draft. JC: Writing – review & editing. BL: Writing – review & editing. JT: Writing – review & editing. YS: Visualization, Writing – review & editing. JJ: Formal analysis, Writing – review & editing. ED: Conceptualization, Writing – review & editing. JW: Conceptualization, Funding acquisition, Writing – review & editing. HL: Conceptualization, Funding acquisition, Supervision, Writing – review & editing.
