# Supplementary material for: A ferritin nanoparticle vaccine based on the hemagglutinin extracellular domain of swine influenza A (H1N1) virus elicits protective immune responses in mice and pigs
Source: Front Immunol. 2024 May 21;15:1361323. doi: 10.3389/fimmu.2024.1361323 (PMC11148206; doi:10.3389/fimmu.2024.1361323)
Supplement: Supplementary file 1 [file DataSheet_1.zip › Raw Data/Body Temperature after challenge.docx]

Table 3 Rectal temperatures of immunized piglets after challenge with swine influenza virus H1N1.

| Days Groups | PBS | IIV | HA-Ferritin | HA-Ferritin+MF59 | NC |
| --- | --- | --- | --- | --- | --- |
| 0d | 39.8/39.8/39.8 (℃) | 39.8/39.8/39.8/39.6/39.7 (℃) | 39.8/39.8/39.8/39.6/39.8 (℃) | 39.6/39.5/39.8/39.6/39.7 (℃) | 39.8/39.8 (℃) |
| 1d | 40.4/40.5/40.3 (℃) | 40.2/40.1/40.3/40.0/40.2 (℃) | 40.2/40.3/40.3/40.2/40.1 (℃) | 39.9/40.0/39.8/39.7/39.8 (℃) | 39.7/39.8 (℃) |
| 2d | 40.8/40.8/41.0 (℃) | 40.2/40.1/39.9/40.1/40.1 (℃) | 39.8/39.9/39.9/39.6/39.5 (℃) | 39.8/39.9/39.9/39.6/39.5 (℃) | 39.7/39.9 (℃) |
| 3d | 40.6/40.9/41.0 (℃) | 40.1/39.9/39.8/39.9/40.2 (℃) | 39.6/39.7/39.8/39.9/39.7 (℃) | 39.6/39.7/39.8/39.7/39.7 (℃) | 39.7/39.6 (℃) |
| 4d | 40.5/40.8/40.7 (℃) | 40.1/40.0/39.9/40.0/39.8 (℃) | 39.8/39.6/39.7/39.7/39.6 (℃) | 39.7/39.6/39.7/39.6/39.7 (℃) | 39.8/39.7 (℃) |
| 5d | 40.4/40.5/40.5 (℃) | 39.5/39.9/39.8/39.8/39.6 (℃) | 39.5/39.7/39.8/39.6/39.8 (℃) | 39.5/39.7/39.8/39.6/39.8 (℃) | 39.8/39.8 (℃) |
| 6d | 40.4/40.5/40.3 (℃) | 40.1/39.9/39.6/39.9/40.0 (℃) | 39.6/39.8/39.8/39.6/39.6 (℃) | 39.6/39.6/39.8/39.6/39.6 (℃) | 39.6/39.8 (℃) |
