# Supplementary material for: A ferritin nanoparticle vaccine based on the hemagglutinin extracellular domain of swine influenza A (H1N1) virus elicits protective immune responses in mice and pigs
Source: Front Immunol. 2024 May 21;15:1361323. doi: 10.3389/fimmu.2024.1361323 (PMC11148206; doi:10.3389/fimmu.2024.1361323)
Supplement: Supplementary file 1 [file DataSheet_1.zip › Raw Data/HAI-mice.docx]

Table 1 Results of hemagglutination inhibition in mice.

| Groups Days | 14d | | | | | | | | 35d | | | | | | | |
| --- | --- | --- | --- | --- | --- | --- | --- | --- | --- | --- | --- | --- | --- | --- | --- | --- |
| PBS | 2^2^ | 2^3^ | 2^2^ | 2^2^ | 2^2^ | 2^2^ | 2^3^ | 2^2^ | 2^2^ | 2^3^ | 2^2^ | 2^2^ | 2^3^ | 2^2^ | 2^2^ | 2^3^ |
| Ferritin | 2^2^ | 2^3^ | 2^2^ | 2^3^ | 2^2^ | 2^2^ | 2^2^ | 2^3^ | 2^2^ | 2^2^ | 2^2^ | 2^3^ | 2^3^ | 2^2^ | 2^2^ | 2^3^ |
| IIV | 2^5^ | 2^5^ | 2^5^ | 2^6^ | 2^5^ | 2^5^ | 2^6^ | 2^5^ | 2^7^ | 2^7^ | 2^7^ | 2^8^ | 2^7^ | 2^8^ | 2^7^ | 2^7^ |
| HANP | 2^6^ | 2^5^ | 2^6^ | 2^6^ | 2^7^ | 2^5^ | 2^5^ | 2^6^ | 2^7^ | 2^8^ | 2^8^ | 2^8^ | 2^7^ | 2^7^ | 2^8^ | 2^6^ |
| HA-Ferritin | 2^7^ | 2^7^ | 2^7^ | 2^8^ | 2^7^ | 2^7^ | 2^7^ | 2^7^ | 2^10^ | 2^9^ | 2^10^ | 2^9^ | 2^10^ | 2^10^ | 2^8^ | 2^10^ |
| HA-Ferritin+MF59 | 2^7^ | 2^8^ | 2^7^ | 2^8^ | 2^7^ | 2^8^ | 2^8^ | 2^9^ | 2^12^ | 2^11^ | 2^12^ | 2^11^ | 2^10^ | 2^12^ | 2^12^ | 2^12^ |
