# Supplementary figures and images for: A ferritin nanoparticle vaccine based on the hemagglutinin extracellular domain of swine influenza A (H1N1) virus elicits protective immune responses in mice and pigs
Source: Front Immunol. 2024 May 21;15:1361323. doi: 10.3389/fimmu.2024.1361323 (PMC11148206; doi:10.3389/fimmu.2024.1361323)

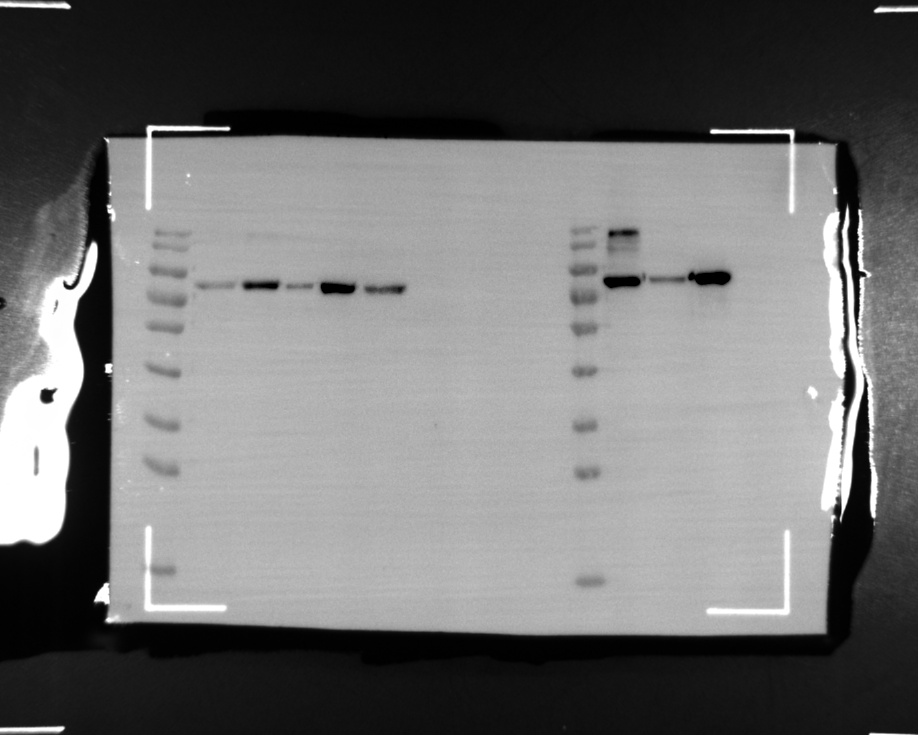

Supplement: Supplementary file 1 [file DataSheet_1.zip › Raw Data/Figure 1B- WB.jpg]

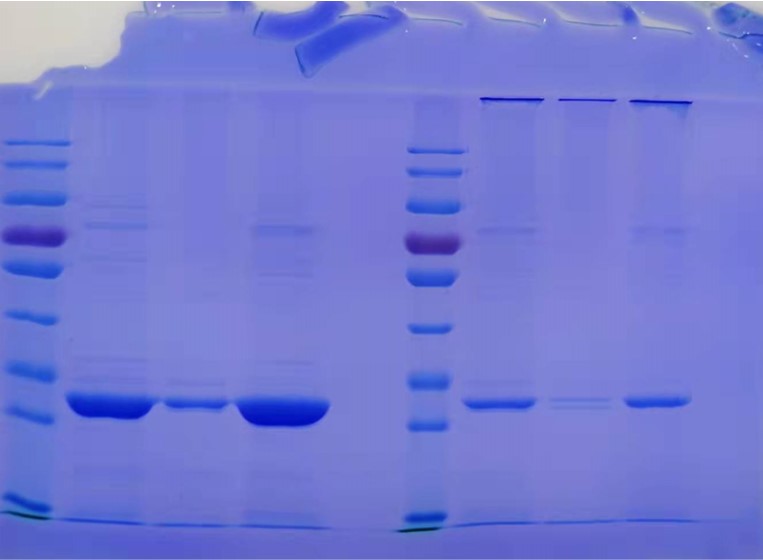

Supplement: Supplementary file 1 [file DataSheet_1.zip › Raw Data/Figure 1D- SDS-PAGE-Ferritin.jpg]

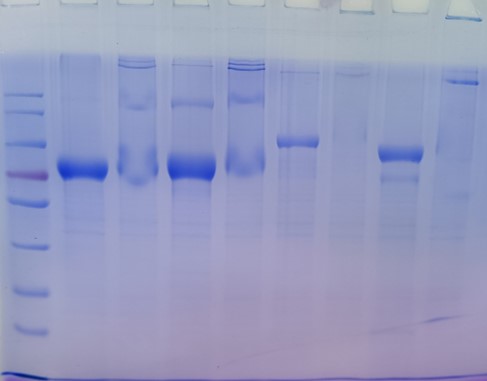

Supplement: Supplementary file 1 [file DataSheet_1.zip › Raw Data/Figure 1D- SDS-PAGE-HA-Ferritin.jpg]
